# Dentoalveolar Complex Autotransplantation in Esthetic Zone Rehabilitation: A Case Report

**DOI:** 10.1155/crid/2931715

**Published:** 2026-02-18

**Authors:** Gurgen V. Khachatrian, Jaime L. Lozada, Robert A. Handysides, Ekaterina A. Zernitckaia

**Affiliations:** ^1^ Private Practice, Tomsk, Russia; ^2^ Advanced Education Program in Implant Dentistry, Loma Linda University School of Dentistry, Loma Linda, California, USA, llu.edu; ^3^ Department of Endodontics, Loma Linda University School of Dentistry, Loma Linda, California, USA, llu.edu; ^4^ Implant Dentistry, Loma Linda University School of Dentistry, Loma Linda, California, USA, llu.edu

## Abstract

Tooth autotransplantation is a biologically favorable option for the replacement of missing teeth and has demonstrated predictable outcomes with both immature and mature donor teeth. In challenging anterior maxillary defects, digitally assisted variations of the technique may expand treatment possibilities and offer an alternative to conventional bone augmentation in selected cases. This article presents a digital and clinical workflow for dentoalveolar complex (DAC) autotransplantation in anterior maxillary reconstruction. The reported case involves transplantation of a donor tooth together with its surrounding alveolar bone segment to compensate for a severe site deficiency. Virtual planning, guided osteotomy, and 3D‐printed replicas were used to optimize donor tooth assessment, recipient site preparation, and extraoral handling efficiency. These digital tools facilitated accurate positioning of the DAC and improved procedural predictability. The case demonstrated uneventful healing, preservation of soft and hard tissue contours, and no clinical signs of ankylosis or significant replacement resorption during follow‐up. This report suggests that digitally guided DAC autotransplantation may represent a viable treatment option for selected anterior defects where conventional approaches, including bone grafting or implant therapy, present biological or anatomical limitations.

## 1. Introduction

Tooth loss in the anterior maxilla presents a multifactorial challenge that often includes alveolar bone resorption and soft tissue collapse. These deficiencies may arise from trauma, congenital anomalies, inflammatory disease, or complications following oncologic and surgical interventions. Reconstructive options typically involve bone augmentation procedures using autogenous grafts, allogeneic materials, xenografts, or synthetic substitutes [1, 2]. Although these approaches can provide predictable outcomes, they are associated with donor site morbidity, increased treatment duration, graft resorption, and the need for staged interventions, which may be particularly problematic in young patients or in cases with extensive defects.

Tooth autotransplantation, defined as the surgical relocation of a tooth within the same individual, offers a biologically favorable option in selected indications [3, 4]. When appropriate case selection and atraumatic handling of the periodontal ligament are respected, long‐term survival and success rates exceeding 85%–90% have been reported [5–9].

Classically, donor selection in autotransplantation has favored teeth with incomplete root formation because this condition has been associated with a higher probability of spontaneous pulp revascularization. However, pulp healing in mature donor teeth has also been described, and both clinical and radiographic success has been reported when atraumatic extraction, short extraoral time, and appropriate endodontic strategies are applied [10, 11]. In addition, while conventional protocols often assume a recipient site capable of accommodating the transplanted tooth without major modification, the transplanted tooth itself can contribute significantly to alveolar regeneration. Autotransplantation has been used to close oral sinus communications and to regenerate buccal bone walls, sometimes in combination with root segment techniques [12, 13].

In response to these challenges, the concept of dentoalveolar complex (DAC) autotransplantation has emerged. This approach involves the transfer of a tooth along with its surrounding alveolar bone and associated soft tissues as a biological unit. By maintaining the native integrity of the periodontal ligament, periosteum, and bone architecture, DAC transplantation facilitates enhanced revascularization, accelerated integration, and immediate restoration of hard and soft tissue volume. This strategy may reduce the need for extensive bone grafting procedures in selected defects and offers a single stage approach to both tooth replacement and ridge reconstruction [14].

Clinical reports have described the use of dentoalveolar segment transplantation for the management of impacted teeth, cleft associated defects, and anterior maxillary deficiencies with high esthetic demands [14, 15]. The potential of these techniques to support maintenance of alveolar dimensions is of particular interest in adolescent and young adult patients, in whom conventional implant therapy must often be delayed.

Despite these promising reports, DAC autotransplantation remains underrepresented in the literature, and no standardized digital or surgical protocol has yet been established. The purpose of this case report is to describe the clinical application of a digitally guided DAC autotransplantation strategy in a patient with a severe anterior maxillary defect. This report illustrates the transplantation of a donor tooth together with its surrounding alveolar bone segment to compensate for pronounced site deficiency. The treatment was performed using three‐dimensional digital planning, 3D‐printed models, and guided osteotomies within an interdisciplinary workflow. By outlining a step‐by‐step clinical protocol and discussing the indications, advantages, and limitations of this approach, this case is aimed at increasing awareness of DAC‐based reconstruction as a potential alternative or adjunct to conventional bone grafting and implant‐supported rehabilitation in selected anterior defects.

## 2. Case Description

This case report describes the clinical application of autogenous DAC transplantation in a patient with a significant anatomical challenge. All procedures were performed by the same surgical team following a strict protocol with preoperative CBCT, CAD/CAM modeling, and postoperative follow‐up.

In addition to the clinical steps performed in the reported case, this section also presents a theoretical protocol for DAC transplantation. The considerations regarding endodontic management and crown reshaping are based on established clinical guidelines and current literature and are included to provide a comprehensive framework for clinical decision‐making.

### 2.1. Preoperative Diagnostics and Planning

A comprehensive diagnostic protocol is essential to confirm feasibility and anticipate esthetic and functional outcomes. Clinical assessment includes evaluation of the periodontal biotype, mucosal thickness, keratinized tissue, and standardized photographic documentation. Panoramic and periapical radiographs provide preliminary anatomical detail, while CBCT scans are mandatory for 3D assessment of ridge volume, bone density, and proximity to critical structures.

Digital planning integrates CBCT and intraoral scans for virtual modeling of donor and recipient sites. Customized surgical guides are fabricated via 3D printing to enhance osteotomy precision and reproducibility. Donor selection typically involves premolars, third molars, or any tooth with suitable root morphology and intact surrounding periodontal tissues. Interdisciplinary input from orthodontists, prosthodontists, and periodontists guides timing, occlusal design, and adjunctive soft tissue management.

### 2.2. Surgical Protocol Overview

Recipient bed preparation includes flap elevation, debridement, antiseptic irrigation, and contouring under guidance of surgical models or templates. Accurate three‐dimensional positioning of the transplanted DAC is fundamental to achieving predictable integration and long‐term success. The spatial alignment of the DAC influences not only osseointegration and primary stability but also the surrounding soft tissue architecture, esthetic harmony, and functional outcomes.

• Vertical positioning requires that the root be placed approximately 1–2 mm apical to the projected cementoenamel junction (CEJ) of adjacent teeth. This minor submersion supports the reestablishment of biologic width and promotes a natural gingival contour, particularly in the esthetic zone.
• Buccolingual placement should position the transplant centrally within the alveolar ridge, with a slight palatal or lingual inclination when indicated. This approach helps preserve the thickness of the buccal bone plate, minimizing the risk of dehiscence or resorption and enhancing long‐term esthetic stability.
• Mesiodistal spacing must allow a minimum clearance of 0.5 mm from adjacent roots to ensure an adequate periodontal ligament interface and to prevent encroachment on neighboring structures.
• Axial alignment should replicate the long axis of the natural predecessor tooth to ensure favorable load distribution and avoid functional overload or angular stress, which can compromise periodontal adaptation.
• Socket fit should be intimate, although a peripheral gap of up to 2 mm is clinically acceptable. In cases of larger discrepancies, the use of osteoconductive materials or autogenous bone chips is advised to facilitate osseous bridging and spatial stability.
• Fixation may be achieved with titanium screws, resorbable pins, or splints depending on graft morphology and bone quality.

Tension‐free soft tissue closure is obtained with periosteal release and layered suturing using monofilament materials; in cases of deficiency, a connective tissue graft may be added.

• Soft tissue considerations include aligning the CEJ of the DAC with the natural curvature of the arch to support harmonious gingival emergence. Excessive submergence (>2 mm) may predispose to soft tissue hypertrophy, recession, or esthetic disharmony.
• Fixation protocols mandate temporary stabilization of the graft for 2–4 weeks, typically with a flexible splint or microfixation system, to maintain positional stability during the critical early healing phase and allow revascularization.
• Radiographic assessment must be performed immediately postoperatively to verify correct positioning, root parallelism, and the absence of socket or cortical plate perforation. This serves as a baseline for future follow‐up and confirms the anatomical fidelity of the transplantation.

### 2.3. Postoperative Management

Postoperative care includes antibiotics (amoxicillin/clavulanate 875/125 mg twice daily for 5–7 days; clindamycin 300 mg for penicillin‐allergic patients), analgesics as needed, and chlorhexidine rinses initiated after the third postoperative day for 1 week. Patients follow a soft diet and avoid mechanical trauma to the site for 2 weeks. Clinical follow‐up is performed at 1, 2, and 4 weeks, with radiographs obtained at 1, 3, 6, and 12 months. Long‐term annual reviews include assessment of graft integration, pulp vitality (if applicable), and periodontal health.

Endodontic therapy is initiated 2–4 weeks postoperatively in teeth with closed apices, while immature teeth are monitored clinically and radiographically to allow for potential revascularization and continued root development. A positive response to electric pulp testing (EPT) is considered indicative of pulp revascularization and continued vitality, supporting a conservative observation approach. Prosthetic rehabilitation is considered at 3–6 months once graft stability is confirmed. Options range from composite restorations to full‐coverage crowns depending on structural integrity. Interim restorations are often employed to guide soft tissue maturation and occlusion. Definitive materials, including lithium disilicate or zirconia, are selected based on esthetic and functional requirements.

### 2.4. Case Presentation

#### 2.4.1. Chief Complaint and Medical History

**Figure 1:**
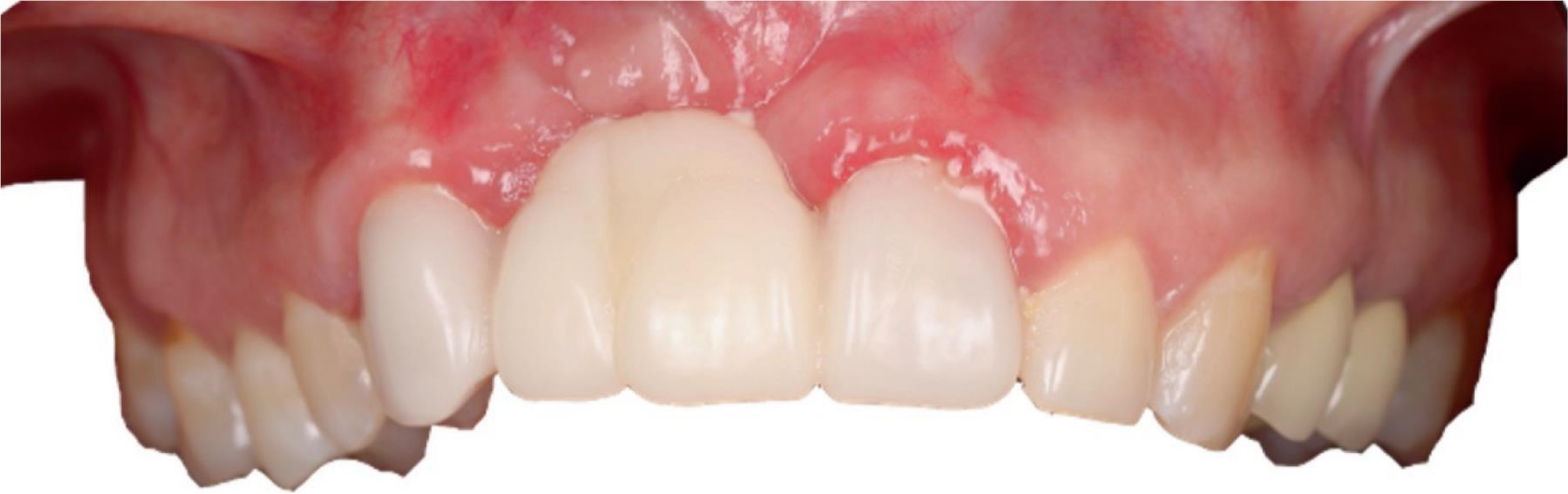
Initial situation with temporary prosthesis supported by Teeth 1.3–2.1.

A 37‐year‐old female patient presented with concerns regarding the absence of maxillary anterior teeth and dissatisfaction with the esthetic outcome of a temporary prosthesis. Her history included four previous unsuccessful interventions performed at another clinic, including two bone augmentation procedures and two implant placement attempts in the regions of Teeth 1.2 and 1.1, all of which failed (Figure 1).

#### 2.4.2. Clinical Examination

**Figure 2:**
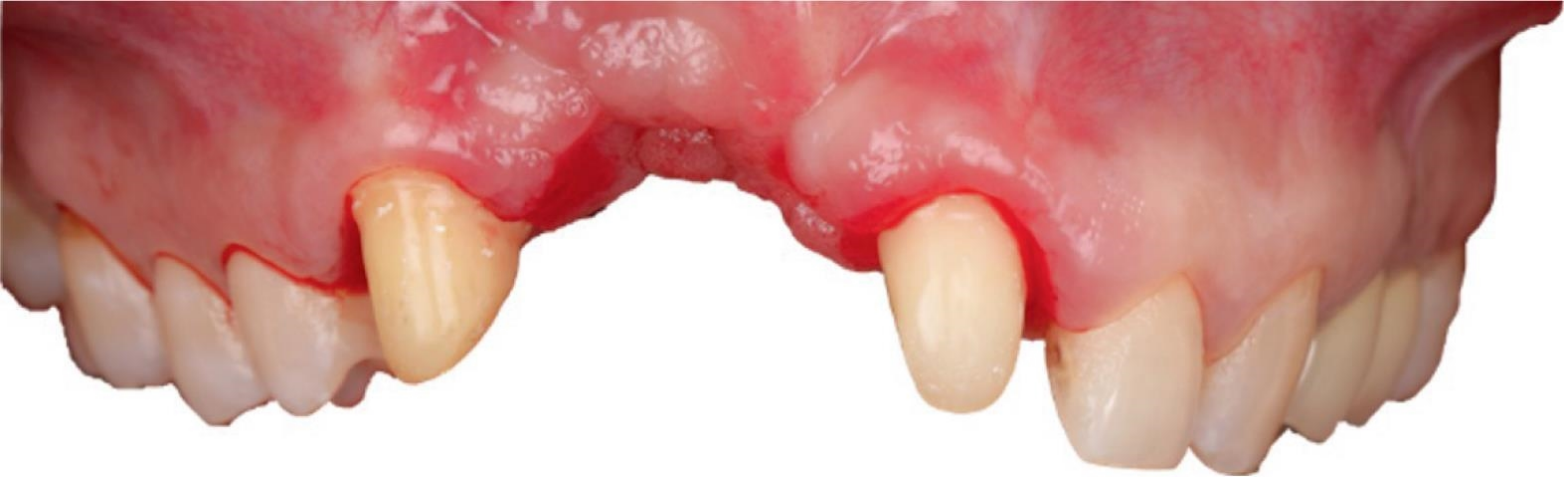
Bone defect visualization after removal of temporary restorations.

Removal of the temporary prosthesis revealed a pronounced horizontal and vertical defect in the area of Teeth 1.2 and 1.1 (Figure 2).

**Figure 3:**
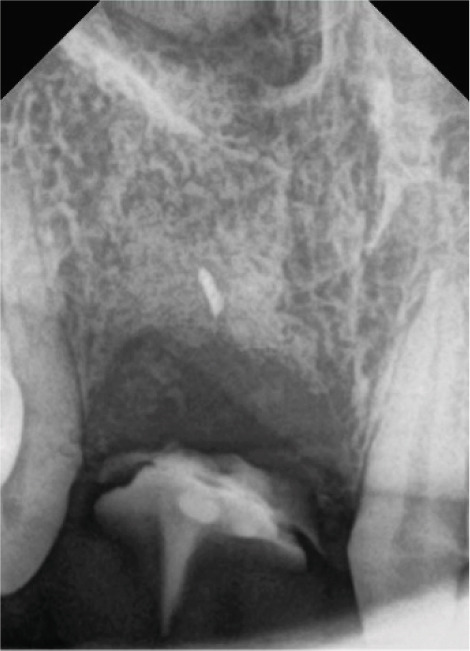
Periapical radiograph before treatment.

CBCT was used to evaluate bone volume and confirmed a combined ridge deficiency, while a periapical radiograph was used to rule out inflammatory pathology and assess root morphology (Figure 3).

#### 2.4.3. Treatment Plan

**Figure 4:**
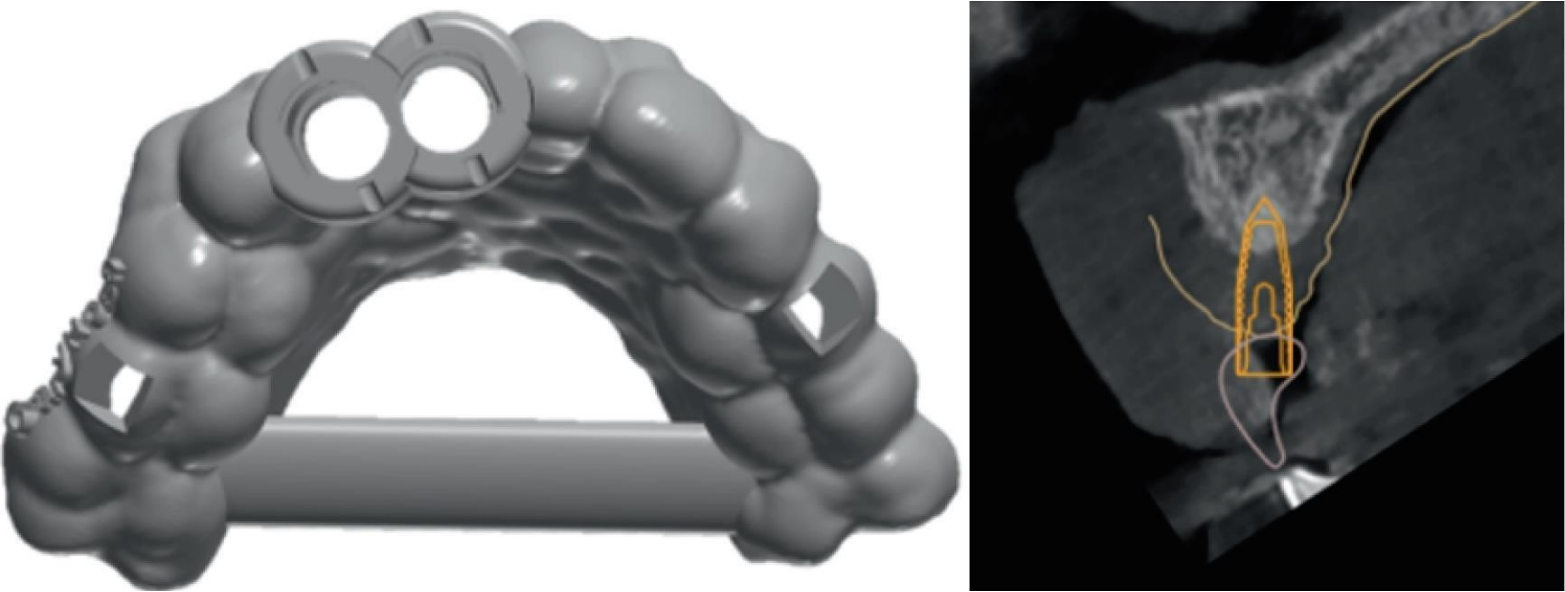
Digitally designed surgical guide for osteotomy preparation.

Given the severe ridge deficiency and the patient′s high esthetic expectations, DAC autotransplantation was selected as a treatment option. Both maxillary third molars (1.8 and 2.8) were evaluated as potential donor sites. Intraoral scans were obtained with the Primescan system and integrated into coDiagnostiX for guided osteotomy planning and fabrication of a custom surgical guide (Figure 4).

**Figure 5:**
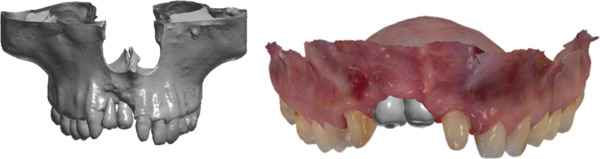
3D‐printed donor tooth models.

STL files of teeth 1.8 and 2.8 were exported from Diagnocat software and printed using a 3D printer (Rapidshape P50 Straumann) to assess spatial compatibility and simulate the transplant (Figure 5).

**Figure 6:**
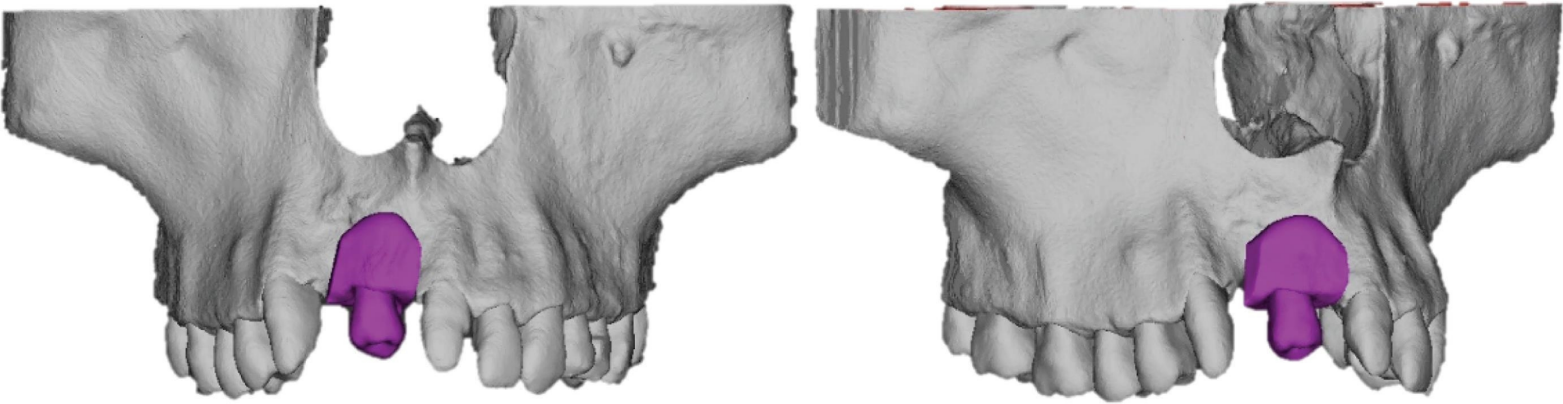
Virtual simulation of DAC transplantation into Site 1.1.

A virtual DAC simulation was performed using Tooth 1.8 as a potential donor (Figure 6). This was intentionally planned as a contingency in the event that Tooth 2.8 proved unsuitable intraoperatively. This planning strategy allowed for assessment of morphological fit, recipient‐site feasibility, and the need for additional osteoplasty, ensuring flexibility during the surgery.

#### 2.4.4. Surgical Stage

**Figure 7:**
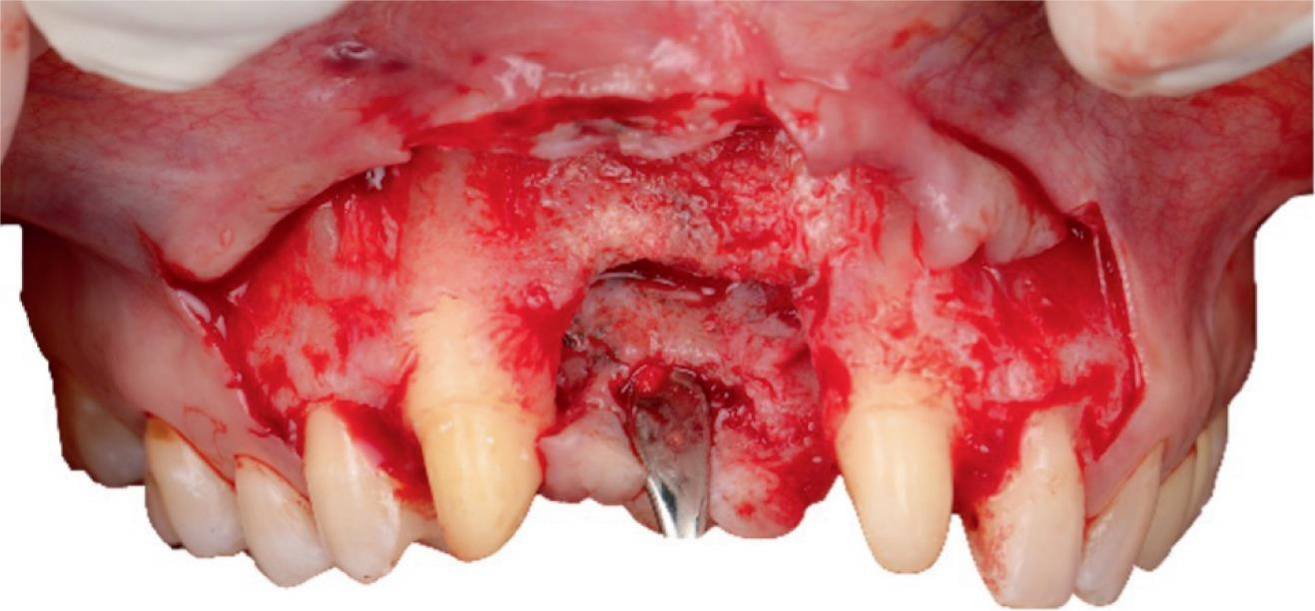
Flap elevation providing access to the recipient site.

Palatal and vertical releasing incisions were made, followed by full thickness mucoperiosteal flap elevation to ensure surgical access and visibility (Figure 7).

**Figure 8:**
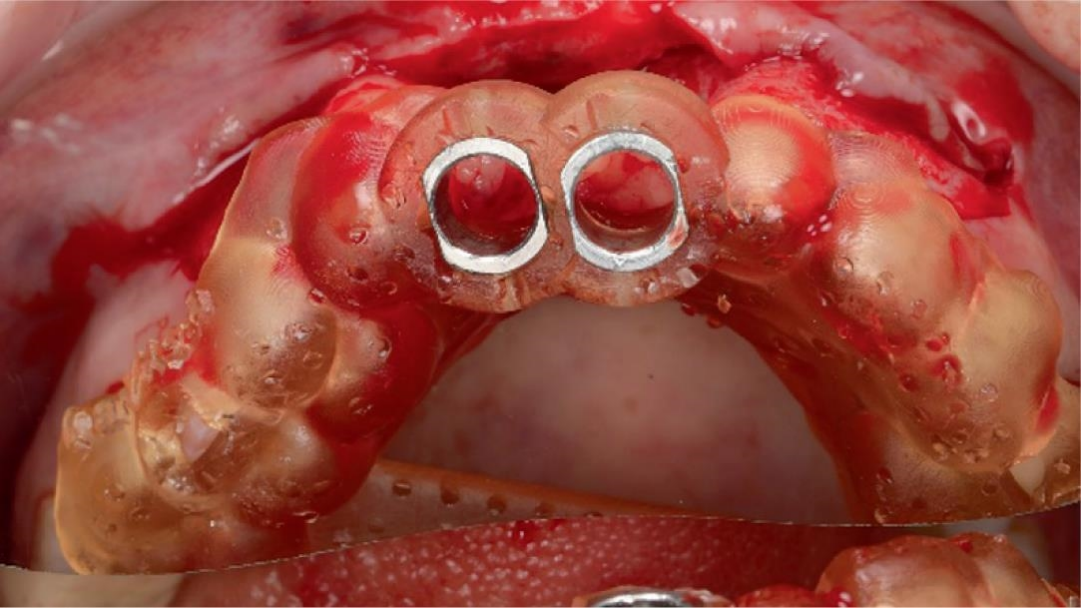
Intraoperative use of the surgical guide for precise osteotomy.

Recipient site preparation was guided using the printed surgical template to ensure the planned angulation and depth (Figure 8).

**Figure 9:**
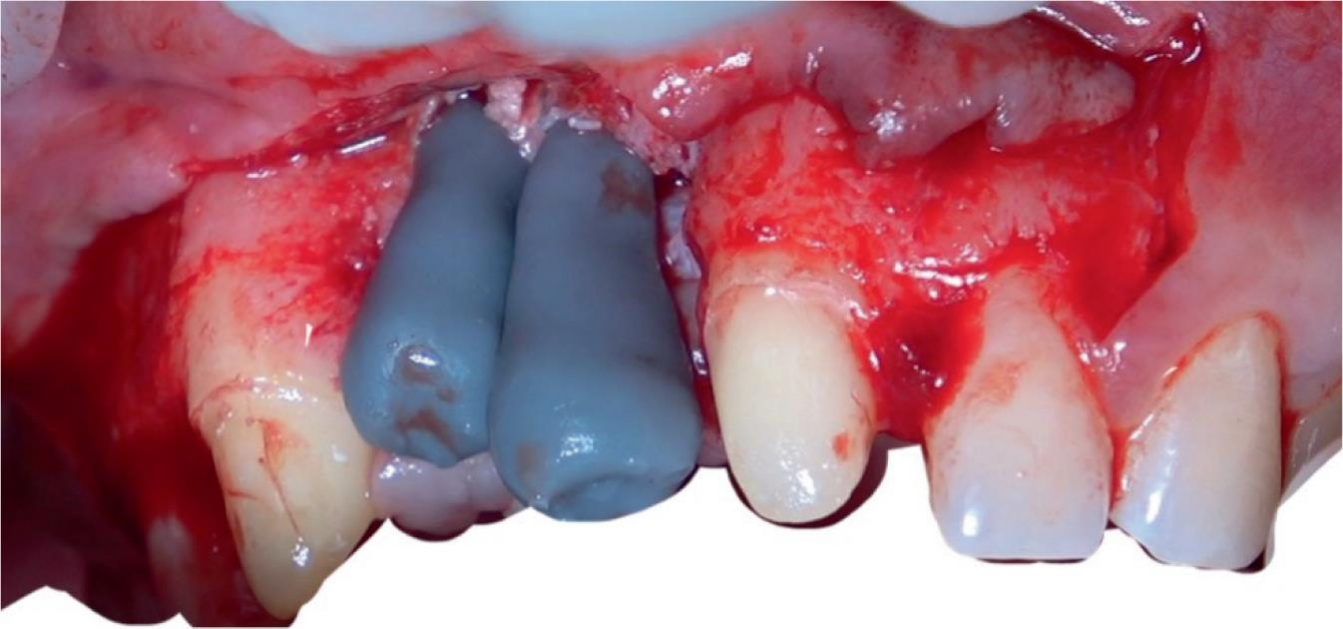
3D‐printed models.

**Figure 10:**
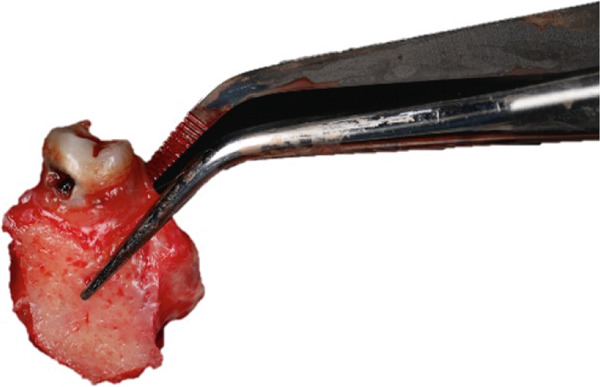
Atraumatic harvesting of DAC in the area of 1.8.

After completion of the osteotomy phase, the 3D‐printed models were trial‐fitted in the defect site (Figure 9). During this intraoperative assessment, it became evident that the available space between the planned recipient sites was more limited than anticipated. The dentoalveolar segment containing Tooth 1.8 was harvested atraumatically using a curved chisel (Figure 10) [16]. Trial insertion demonstrated excellent adaptation of the DAC. Based on this finding, the surgical plan was modified, and transplantation was limited to a single DAC rather than proceeding with reconstruction of two adjacent sites.

**Figure 11:**
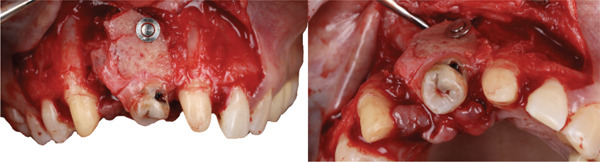
DAC stabilization with a titanium tag at Site 1.1.

**Figure 12:**
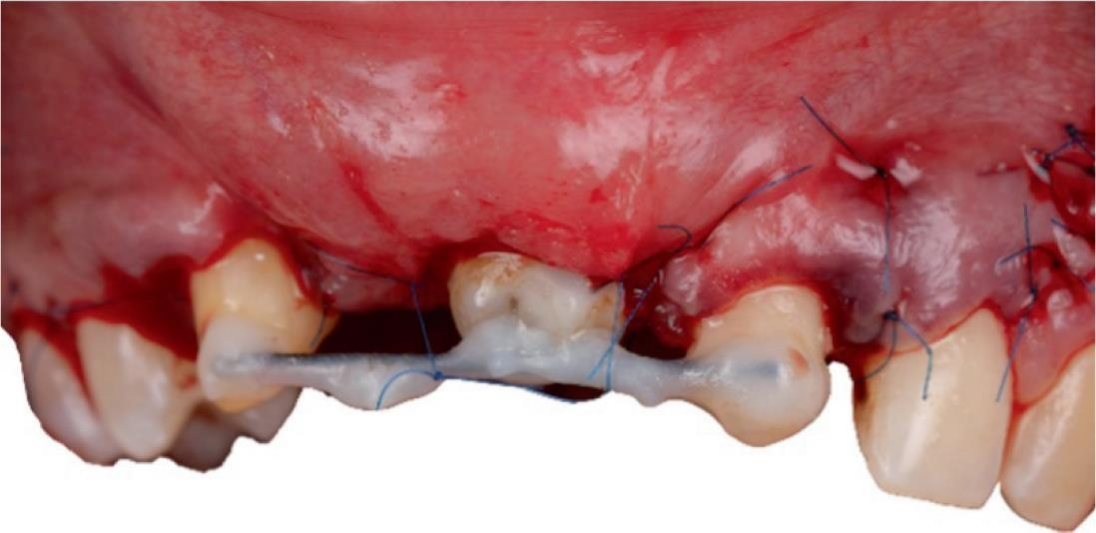
Additional immobilization achieved with wire splinting.

**Figure 13:**
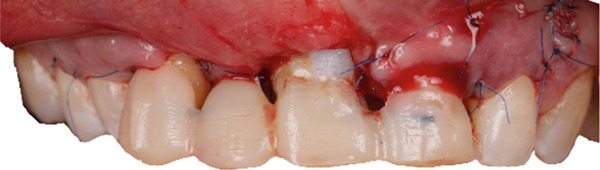
Direct provisional restorations.

The graft was placed into the prepared recipient bed and stabilized using a press‐fit technique in combination with a titanium impact pin (Figure 11). Additional immobilization was achieved with splinting to adjacent teeth using a wire retainer (Figure 12). Soft tissues were mobilized and closed in layers under moderate tension. Direct provisional crowns were fabricated and seated chairside (Figure 13).

**Figure 14:**
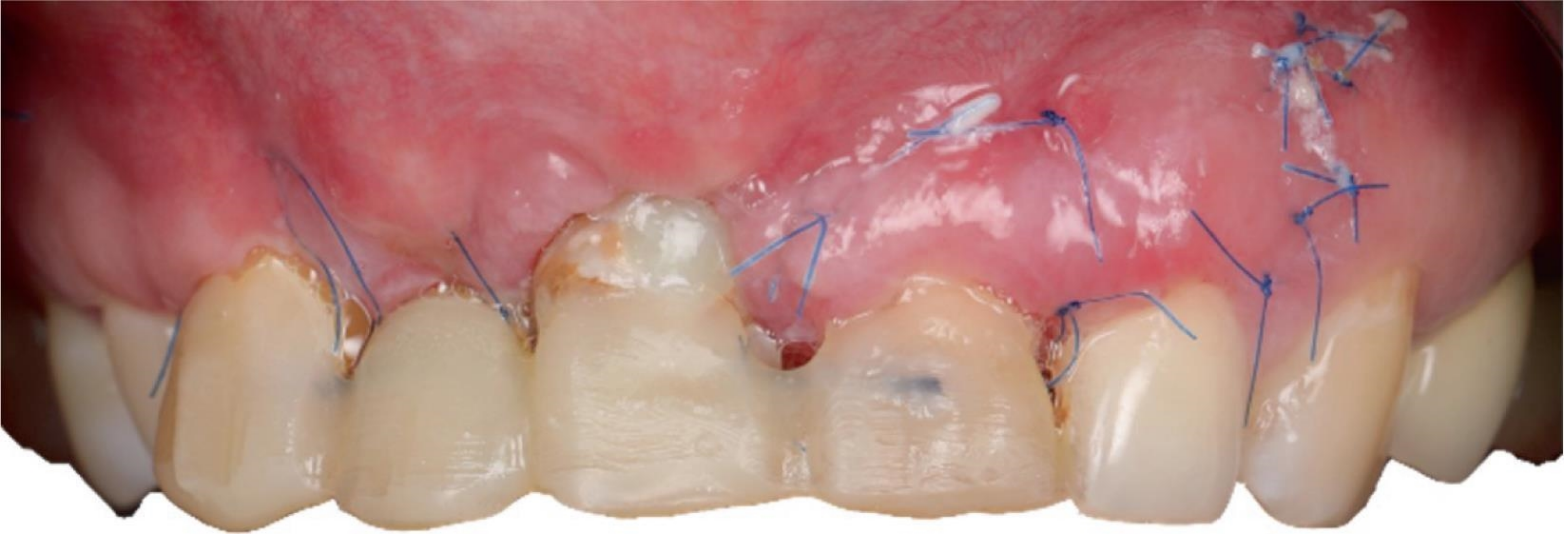
Follow up at 14 days showing soft tissue healing.

The patient received postoperative antibiotics, antiseptic rinses, and analgesics. Instructions included soft diet, limited function, and meticulous hygiene. Soft tissue healing was uneventful, and sutures were removed at 14 days (Figure 14).

**Figure 15:**
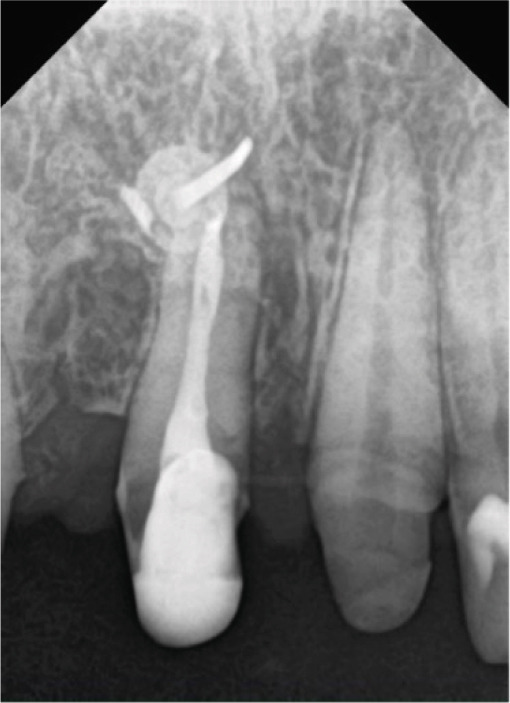
Provisional crown fixed after endodontic treatment.

At 1 month, endodontic treatment of the transplanted tooth was performed, and a provisional crown was placed (Figure 15).

**Figure 16:**
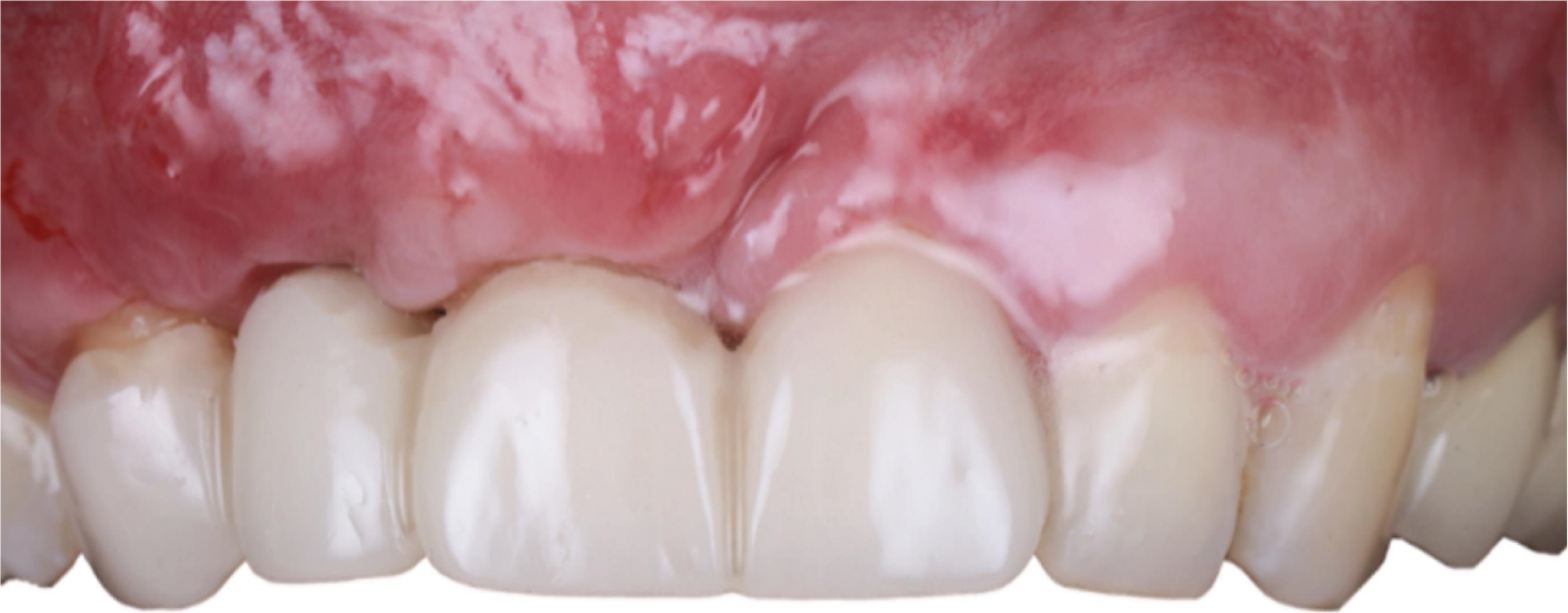
Provisional PMMA crowns after soft tissue augmentation.

At 3 months, soft tissue augmentation of the anterior maxilla and clinical crown lengthening of Teeth 1.2 to 2.3 were performed to harmonize gingival zeniths. Provisional PMMA restorations were placed (Figure 16).

**Figure 17:**
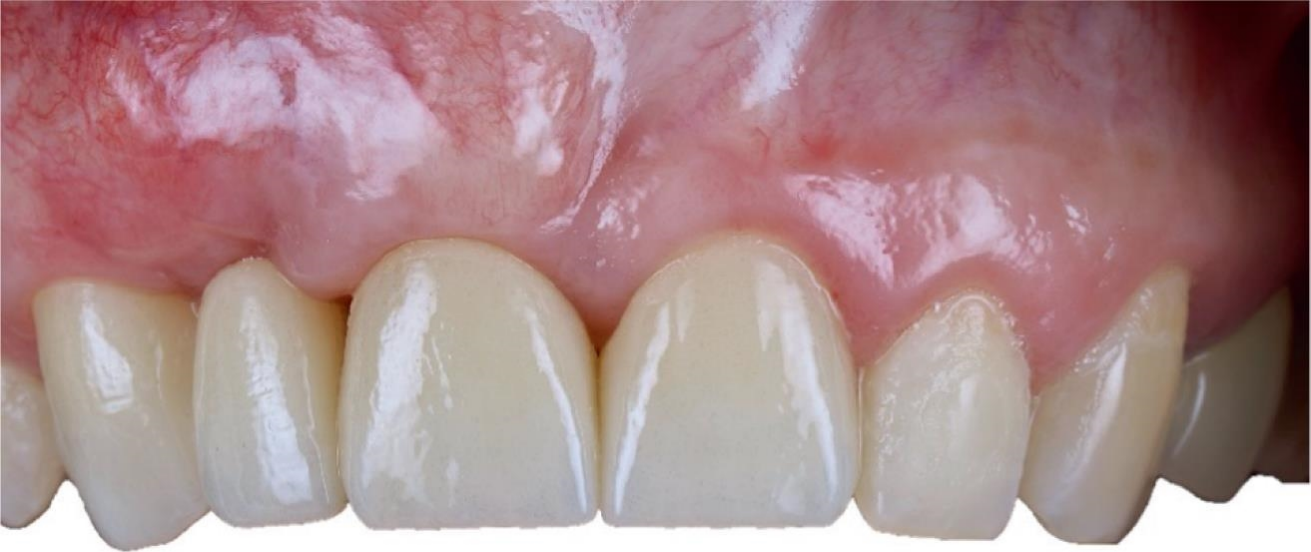
Final prosthetic outcome with zirconia restorations.

**Figure 18:**
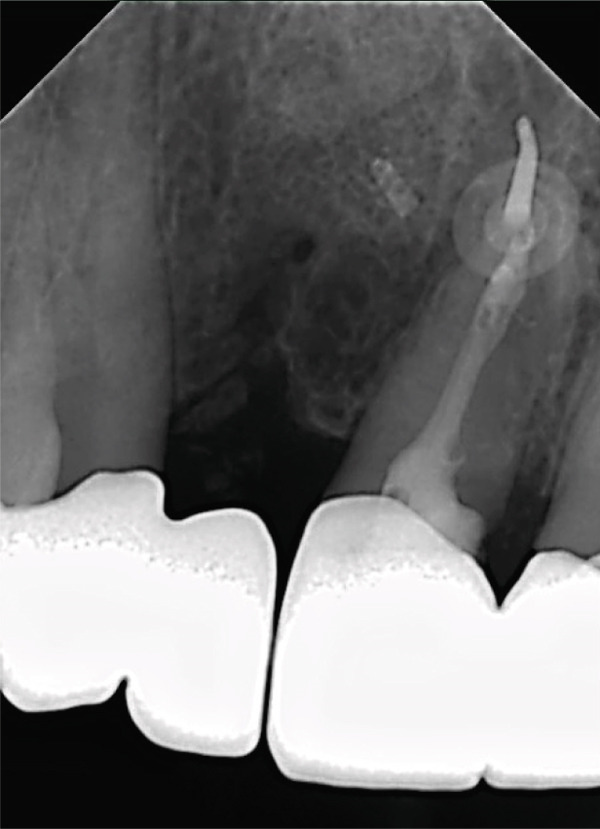
Radiographic evaluation demonstrating osseointegration and transplanted DAC stability.

At 4 months, final prosthetic rehabilitation was completed using zirconia crowns: a cantilever bridge from 1.3 to 2.2 and individual crowns for Teeth 1.2 and 1.1 (Figure 17). A periapical radiograph was taken to confirm stability and osseointegration (Figure 18).

## 3. Discussion

Autotransplantation represents a biologically favorable treatment option for selected cases of tooth loss in both anterior and posterior regions, especially when implant therapy is contraindicated or when prior reconstructive approaches have been unsuccessful. The incorporation of a bone segment with the donor tooth, as performed in DAC transplantation, represents a developing variation of the technique that may broaden reconstructive possibilities in sites with significant anatomical compromise.

In the present report, DAC autotransplantation was applied as a reconstructive strategy to address a severe anterior maxillary defect. This approach provided both hard and soft tissue support in a situation where conventional bone augmentation alone would have been limited by anatomical and clinical constraints. The case illustrates that DAC‐based reconstruction may offer a valuable alternative in selected anterior defects, expanding the therapeutic options when traditional bone grafting or implant placement is not ideal.

Preservation of the periodontal ligament and minimization of extraoral time remain essential biological determinants of success, as described by Tsukiboshi et al. [5, 17]. The DAC technique supports these principles through meticulous surgical planning aided by CBCT‐based imaging, digital simulations, and the fabrication of 3D‐printed transplant replicas and surgical guides. This workflow enables high‐precision osteotomy preparation, reduces intraoperative trauma, and enhances the accuracy of transplant positioning, factors that are critical in maintaining periodontal ligament vitality and preventing root resorption or ankylosis.

While en bloc transplantation methods, such as those described by Krasny et al., have demonstrated excellent outcomes in the management of impacted teeth and anterior maxillary defects, they often involve the transfer of a large bone volume, which can increase surgical morbidity [14, 18]. In contrast, DAC transplantation involves a more conservative, yet biologically strategic, harvest of the donor site. This approach is aimed at preserving essential regenerative elements while reducing the invasiveness of the procedure. By transferring a unit tailored to the recipient anatomy, DAC transplantation achieves an optimal balance between biological preservation and surgical practicality.

Moreover, DAC transplantation may serve as a biologically advantageous alternative to GBR in select cases [1, 2]. While GBR remains a widely accepted and effective approach for alveolar ridge augmentation, it often involves the use of alloplastic or xenogeneic materials and requires extended healing periods. In contrast, DAC provides an autogenous, vascularized graft that can restore both bone volume and periodontal function immediately, potentially reducing treatment time and improving tissue stability.

Nevertheless, the technique is not without limitations. DAC transplantation is surgically demanding and necessitates advanced planning, careful patient selection, and technical expertise. Complications such as inflammatory root resorption, pulpal necrosis, and soft tissue dehiscence remain possible, although their incidence may be mitigated through adherence to biological principles and early endodontic management. For teeth with complete root formation, initiation of root canal therapy within 2–4 weeks postoperatively remains critical to reducing the risk of inflammatory complications, as supported by recent clinical studies [7, 19].

In summary, dentoalveolar segment transplantation represents a promising but still developing technique within the broader spectrum of autotransplantation procedures. When supported by digital planning and applied selectively, it may offer a reconstructive option in anterior maxillary defects where conventional approaches present limitations. Further studies with larger cohorts are needed to define indications, refine protocols, and assess long‐term outcomes.

## 4. Conclusion

DAC transplantation, as illustrated in this report, may represent a viable reconstructive option in selected anterior maxillary defects, particularly when conventional bone augmentation is limited by anatomical or clinical constraints. The integration of digital planning, 3D‐printed models, and guided osteotomy facilitates accurate recipient site preparation and supports predictable positioning of the transplanted dentoalveolar segment.

In the presented case, a digitally guided DAC autotransplantation approach was applied to address a complex defect, demonstrating stable healing and satisfactory esthetic and functional outcomes during follow‐up.

Although these findings are encouraging, further clinical studies with larger sample sizes and long‐term follow‐up are needed to better define the indications, limitations, and success parameters of DAC transplantation.

## Funding

No funding was received for this manuscript.

## Conflicts of Interest

The authors declare no conflicts of interest.

## Data Availability

The data that support the findings of this study are available from the corresponding author upon reasonable request.
